# Arthritis Awareness Month — May 2019

**DOI:** 10.15585/mmwr.mm6817a1

**Published:** 2019-05-03

Arthritis Awareness Month, led by the Arthritis Foundation (https://www.arthritis.org), is observed each May to bring attention to arthritis and its impact. Arthritis affects an estimated 54.4 million U.S. adults, or approximately one in four (*1*); and of these adults with arthritis, approximately 27% have severe joint pain (*2*). Arthritis also is linked to higher rates of physical inactivity (*1*).

A report in this issue of *MMWR* found that arthritis is more common among American Indian/Alaska Natives than among any other racial/ethnic group and is most prevalent in Appalachia and the Lower Mississippi Valley regions (*3*). Likewise, the report found that, in all states, severe joint pain and physical inactivity were common among adults with arthritis, but especially among those in southeastern states, and were most common among adults who were disabled or unable to work (*3*). Adults with arthritis and severe joint pain also were more likely to be physically inactive than those with no or mild to moderate joint pain (*3*) even though physical activity eases arthritis pain over time (*1*).* CDC supports evidence-based lifestyle management programs proven to help adults with arthritis to be physically active and improve their quality of life.^†^

## Footnotes

* https://www.cdc.gov/arthritis/basics/physical-activity-overview.html.

† https://www.cdc.gov/arthritis/interventions/physical-activity.html.
